# A cDNA Cloning of a Novel Alpha-Class Tyrosinase of *Pinctada fucata*: Its Expression Analysis and Characterization of the Expressed Protein

**DOI:** 10.1155/2014/780549

**Published:** 2014-04-10

**Authors:** Ryousuke Takgi, Tomoyuki Miyashita

**Affiliations:** Department of Genetic Engineering, Faculty of Biology-Oriented Science and Technology, Kinki University, 930 Nishimitani, Kinokawa, Wakayama 649-6493, Japan

## Abstract

Tyrosinase plays an important role in the formation of the shell matrix and melanin synthesis in mollusks shells. A cDNA clone encoding a 47 kDa protein was isolated from the pearl oyster *Pinctada fucata*. The cDNA was 1,957 base pairs long and encodes a 417 residue protein that has extensive sequence identity with tyrosinase (polyphenol oxidase: EC 1.14.18.1). This tyrosinase-like protein, termed PfTy, contains an N-terminal signal sequence and the two copper-binding domain signatures (CuA and CuB), suggesting that PfTy belongs to the **α**-subclass of type-3 copper proteins. Enzyme activity of PfTy was examined by a spectrophotometric method using the translation product derived from an S30 T7 high-yield protein expression system. Tyrosinase activity was seen in this recombinant product. RT-PCR analysis showed that PfTy mRNA was expressed in the mantle pallial, but not in the mantle edge. Therefore, PfTy may participate in insoluble shell matrix formation of the nacreous layer. PfTy expression was also observed in the foot, liver, and adductor muscle, suggesting that PfTy participates in the synthesis of melanins, which are effective scavengers of free radicals formed in multiple intracellular oxidative processes. This is the first report of a novel **α**-class tyrosinase from the pearl oyster *P. fucata*.

## 1. Introduction

Copper-containing proteins play very important roles in the regulation of the metabolism of an organism [1]. They can be classified into three groups, namely, types 1 to 3, by the geometric structure of the active site and spectroscopic properties [2]. Phenoloxidases (POs) belong to the type 3 group, which includes hemocyanin [2]. POs can be divided into tyrosinases (EC 1.14.18.1), laccases (EC 1.10.3.2), and catecholoxidases (EC 1.10.3.1).

It is well known that tyrosinase (TYR)/polyphenoloxidase (PPO) (EC 1.14.18.1) is an important bifunctional copper-dependent enzyme. This enzyme catalyzes a reaction that converts L-tyrosine to L-DOPA by hydroxylation and then to L-dopaquinone. L-Dopaquinone is spontaneously changed through a series of reactions and then polymerizes to become melanin [3]. The synthesized melanin leads to the observed browning of plants or mushrooms, the coloring of skin, eyes, and hair in humans [4], and the periostracum of mollusks shells [5]. Melanin also participates in the defense system. Synthesized melanin has antipathogenic activity. Most melanins derived from the hemolymph exist as an insoluble form and this form has physicochemical properties similar to defense-related proteins that work as ultraviolet or X-ray absorbers and cation exchangers. As for the antipathogenic activity of the insoluble form of melanin, the synthesis of melanin is observed in the wounds of various invertebrate animals. Melanization is thought to occur through the deposition of melanin on the parasite surface and plays a key role in wound healing and defense against pathogen infections in the invertebrate defense system [6, 7]. In arthropods, a PO oxidizes N-acylcatecholamines to quinones and quinone methides. These products can act as reactive electrophile cross-linking agents between cuticular proteins to form the exoskeleton of arthropods [8].

In the shells of mollusks, a variety of inter- and intramolecular crosslinks are found in proteins, including a quinone derivative, a pair of tyrosyl residues, and a cysteine cross-link, and these crosslinks are responsible for the formation of hard-tissue [9]. Tyrosinase catalyzes the coupling of tyrosyl residues. High tyrosine content is associated with quinone-tanned proteins [10, 11]. It has been suggested that cross-linked polymerization of protein chains may be related to the insolubility of the shell matrix [9, 12]. Recently, three tyrosinase-like genes, which may participate in the reaction of the melanin synthesis and/or the polymerization of quinone-crosslinking of the insoluble shell matrix, were reported in* Pinctada fucata*. They are Pfty1, Pfty2 [13], and OT47 [5].* Pfty1* is expressed in the mantle edge and pallial, whereas* Pfty2 *is expressed only in the mantle pallial. OT47 is expressed only in the mantle edge. Therefore,* Pfty1* and* OT47 *may participate in insoluble shell matrix and/or pigment formation of periostracum and the prismatic layer.* Pfty2 *may participate in the insoluble shell matrix of the nacreous layer.

The pearl oyster* P. fucata* produces two kinds of pearl: that with a black and blue bristle tail or with no tail originating from the presence/absence of a defense-related protein secreted by injured mantle tissue or hemocytes. We extracted proteins from the two types of pearl in a variety of ways and compared the contents of the protein extract. We found a 47 kDa protein that exists specifically in the nacreous layer of the black and blue pearl and possesses tyrosinase/polyphenoloxidase activity as determined by zymography [14]. To isolate the cDNA of the 47 kDa protein, we have screened a cDNA expression library with an antibody against the 47 kDa protein and obtained a cDNA clone that was not equivalent to the 47 kDa protein, judging from the amino acid sequence. However, a homology search of the deduced amino acid sequence showed that this protein has an extensive identity with tyrosinase. In this report, we present the cDNA cloning of the tyrosinase-like gene. The cloned gene protein product produced from an in vitro transcription/translation system was found to have tyrosinase activity. The novel tyrosinase, termed PfTy, had a molecular weight of 47, 333 Da and belongs to the *α*-class tyrosinase, according to the taxonomy that has been proposed recently [2]. PfTy was identified to be very close to the genus* Pinctada *and* I. argentinus* tyrosinases by phylogenetic analysis. In this paper, we report the features of this tyrosinase.

## 2. Materials and Methods

### 2.1. Animals and Materials

The pearl oyster* P. fucata* was obtained from Gokasho Bay (Mie Prefecture, Japan) in August 2011. Mushroom tyrosinase of* Agaricus bisporus* was purchased from the Sigma-Aldrich Corporation (St. Louis, MO, USA) (CAS number 9002-10-2).

### 2.2. Extraction of the 47 kDa Protein

Nacreous layers of* P. fucata* pearls were crushed to a fine powder. The powdered pearls (20 g) were suspended in 100 mL of 0.3 M EDTA (ethylenediaminetetraacetic acid) (pH 8.0) containing 0.01% sodium azide. Subsequently, the suspension was extracted at room temperature under conditions of continuous stirring for 3 days. Soluble and insoluble matrix fractions were separated by centrifugation at 30,000 g for 20 min. The EDTA-insoluble fraction was extracted with 8 M urea at 60°C overnight under conditions of vigorous shaking. The urea-soluble extract was obtained by centrifugation at 30,000 g for 20 min. The supernatant (80 mL) was dialyzed against 3 L of H_2_O with three changes. The dialyzed fraction (300 mL) was lyophilized and then dissolved in 4 mL of 30 mM Tris-HCl (pH 8.0).

### 2.3. Purification and Determination of the Partial Amino Acid Sequence of the 47 kDa Protein

To determine the N-terminal amino acid sequence of this 47 kDa protein, proteins of the urea-soluble fraction dissolved in 30 mM Tris-HCl (pH 8.0) were separated by 12.5% sodium dodecyl sulfate polyacrylamide gel electrophoresis (SDS-PAGE) and blotted onto a polyvinylidene fluoride membrane (Millipore) using a dry blotting system (Nippon Eido). After Ponceau S staining, the band was cut out and then subjected to N-terminal amino acid sequence analysis. However, as the N-terminal amino acid was modified, this approach failed. To purify the 47 kDa protein, an extracted sample was applied to a 12.5% SDS-PAGE. After electrophoresis, the gel was stained with R-250, and then the 47 kDa band was excised. The protein was eluted from the gel in SDS-PAGE buffer using an electroeluter (Bio-Rad, Model 422). The eluate was dialyzed against 30 mM Tris-HCl (pH 8.0) and then concentrated by precipitation with 10 volumes of acetone to remove the residual SDS and dye. To obtain the internal sequence for the protein, the purified 47 kDa protein was cleaved with cyanogen bromide. Generated fragments were separated by HPLC. The longest fragment was selected and subjected to amino acid sequence analysis. The resulting amino acid sequence was KEDYRN. Other fragments could not be used to determine the amino acids sequence unambiguously.

### 2.4. Preparation of Antibodies and the Screening of the Mantle cDNA Library

Rabbit IgG was purified by a Protein A Sepharose column (Bio-Rad) from rabbit antiserum made against the purified 47 kDa protein. It was used as a probe to screen the *λ*ZIPLOX (GIBCO-BRL Cat #15397-029) cDNA libraries. Screening of mantle *λ*ZIPLOX cDNA libraries was performed as described in Miyashita et al. [15].

### 2.5. DNA Sequencing

DNA sequencing was performed as described in Miyashita et al. [15].

### 2.6. In Vitro Transcription and Translation

The tyrosinase-like gene cDNA (Tlg-cDNA) insert was excised from pZL1 (Gibco BRL) with* Eco*RI and* Hind*III and recloned into the pET-29b vector (Novagen). The* Eco*RI site is located in the portion encoding the third and fourth amino acid from the N-terminal end. The Tlg-cDNA was transcribed from a T7 promoter and translated in a S30 T7 high-yield protein expression system (Cat. # L1110: Promega, Southampton, UK), supplemented with 0.1 *μ*g of the plasmid. The transcription and translation reactions were performed according to the manufacturer's instructions. The products were treated with RNaseIII at 37°C for 30 min followed by analysis by SDS-PAGE.

### 2.7. SDS-PAGE

Proteins were subjected to SDS-PAGE on 10% or 12.5% acrylamide gels, as described by Laemmli [16]. Perfect protein marker TM 10–225 KDa (Novagen (Cat. # 69079-3)) was used as protein molecules of known molecular weight.

### 2.8. Zymography of Tyrosinase/Polyphenol Oxidase

Tyrosinase/polyphenol oxidase activity was visualized by DOPA staining after SDS-PAGE. This approach is the zymography method [17]. Aliquots of the enzyme preparations were incubated with fivefold aliquots of the sample buffer (60 mM Tris-HCl (pH 6.8), 2% SDS, 10% glycerol) at 37°C for 30 min and then subjected to 10% SDS-PAGE. After electrophoresis, the gel was washed with 0.1 M phosphate-buffered saline (PBS) (pH 6.8) and incubated in 0.1 M PBS (pH 6.8) containing 5 mM L-DOPA substrate with gentle shaking at 37°C for 1 h.

### 2.9. Phylogenetic Analysis

A phylogenetic analysis was performed based on the alignment of the CuB binding domain of tyrosinases and POs from various kinds of vertebrate and invertebrate animals and* Limulus polyphemus* hemocyanin subunit II. The alignments were generated using the site MAFFT (multiple alignment using fast Fourier transform) version 7 program (http://www.ebi.ac.uk/Tools/services/web_mafft/toolform.ebi) and were subjected to phylogenetic analysisby using the neighbor-joining (NJ) method with 1000 replicate runs of bootstrap values.

### 2.10. Spectrophotometric Assay of Tyrosinase

Tyrosinase catalyzes a reaction that converts L-tyrosine to L-DOPA by hydroxylation and then to L-dopaquinone. Dopachrome is a colored compound with a peak absorbance at 475 nm. The dopachrome assay was performed as described in Lerch and Ettinger [18]. The increase in absorption at 475 nm, due to the formation of dopachrome (*ε*_475_ = 3,600 M^−1^ cm^−1^), was monitored as a function of time. We used a 5 mM sodium phosphate buffer (pH 7.0) containing 0.08 mM CuSO_4_ and 5 mM L-DOPA as the substrate solution. The enzyme reaction was initiated by the addition of 20 *μ*L of enzyme solution into 480 *μ*L of the substrate solution. The reaction was monitored by measuring the change in absorbance at 475 nm derived from the formation of the L-DOPA chrome at 37°C using a Jasco V-550UV/VIS spectrophotometer (JASCO). A blank control with 500 *μ*L of the substrate solution was also prepared.

### 2.11. Extraction of the Total RNA

Total RNA was extracted from the mantle edge, the mantle pallial, foot, liver, and adductor muscle of* P. fucata* by using the TRIzol reagent (Invitrogen, Tokyo, Japan), according to the manufacturer's instructions.

### 2.12. Gene Expression Analysis by RT-PCR

Generation of the first strand cDNA was performed by oligo-dT primed reverse transcription (Superscript II; Invitrogen) using an equal amount of total RNA (2 *μ*g) obtained from different tissues. The generated cDNA was used as the template for PCR, which was catalyzed by TaKaRa Taq DNA polymerase (TaKaRa Biomedical, Otsu, Shiga, Japan). An amount of 200 ng each of cDNA was used as the template for PCR. PCR were performed with Advantage 2 polymerase mix (Clontech) using the following cycle conditions: 95°C for 5 min followed by 30 cycles of 95°C for 30 s, 52°C for 30 s, and 72°C for 1 min and terminated by an incubation at 72°C for 1 min. The following primer pairs were used for the amplification of the DNA fragments of pearlin [15] and nacrein [19] genes, respectively: pearlin-f (5′-ATGACGTGCACACTTCGTTG) and pearlin-r (5′-CTTGTCATACCGTTCATCGA) (706 bp) and nac-f (5′-GTATCTTCATTTGACTGCCC) and nac-r (5′-TATTATCAGGGTGATGTCCC) (707 bp).

Four tyrosinase genes have already been reported, including the gene* PfTy* from* P. fucata*. These are* Pfty1*,* Pfty2* [13], and* P. fucata* tyrosinase [5]. The sequences are highly homologous to each other. However, the terminal DNA sequence of the 3′ flanking region of the gene* PfTy* does not exist in the other three genes. To detect expression of the gene* PfTy* specifically by RT-PCR analysis, two primers were designed within the arrangement of the 3′ flanking region of the gene* PfTy*. Therefore, the following primer pairs were used for the amplification of the DNA fragments of PfTy: pfty-f (5′-ATCGTTGAAAGTGTTCCATAACA) and pfty-r (5′-TGAAAAATAAACACTCATTAATG) (168 bp). Moreover, the following primer pairs were used for the amplification of the DNA fragments of *β*-actin as a control: actin-f (5′-TGTAYGCCTCTGGYCGYACC) and actin-r (5′-CVACRTCRCACTTCATGATGS) (380 bp). Primers for the *β*-actin are described in Suzuki et al. [20]. One-fifth of each PCR product was fractionated on a 1.5% agarose gel and photographed after ethidium bromide staining. The PCR products were subcloned into pBluescript SK II SK (+) (Stratagene, La Jolla, CA) followed by sequencing to confirm the target gene.

## 3. Results and Discussion

### 3.1. Purification of the 47 kDa Protein and Preparation of Antibodies

A fine powder of the nacreous layers of* P. fucata* pearls was extracted with EDTA and the EDTA-insoluble fraction was extracted with 8 M urea, as described in Section 2. An extracted sample was analyzed by SDS-PAGE. Purification of the 47 kDa band was performed as described in Section 2. The rabbit antiserum was made against the purified 47 kDa protein and the rabbit IgG was purified by a Protein A Sepharose column.

### 3.2. Cloning and Molecular Characterization of PfTy Tyrosinase cDNA

**Figure 1:**
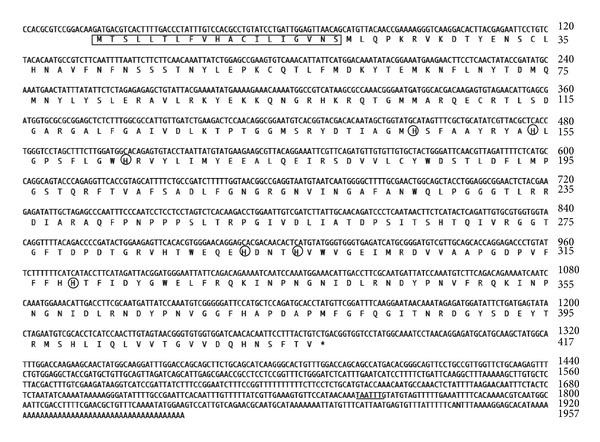
Nucleotide and deduced amino acid sequences of* Pinctada fucata* tyrosinase PfTy. A number of nucleotide and amino acid positions are indicated in the right margin. The putative amino acid sequence of the signal peptide is boxed. The polyadenylation signal TAATTT is underlined with a bold line. The putative histidine copper ligands are circled. The asterisk (∗) in the amino acid sequence indicates the stop codon. GenBank accession number: AB353113.

Anti-rabbit IgG purified by a Protein A Sepharose column was used as a probe to screen *λ*ZIPLOX cDNA libraries. Screening of the mantle *λ*ZIPLOX cDNA libraries was performed as described in Miyashita et al. [15]. About 5 × 10^5^ recombinant phages were screened and five clones that produced strong signals with the antibody were isolated. These cDNA inserts were subcloned into the pBluescript-sequencing vector and then sequenced. The deduced amino acid sequences revealed that two of the inserts had the proper open reading frame and encoded an identical protein. The other three inserts lacked the 3′ end. Therefore, we analyzed the identical clone. The details of the cDNA structure are shown in Figure 1. The cDNA is 1,957 base pairs long. The open reading frame starts at ATG 18 and extends to the stop codon, TGA, at 1267. The open reading frame, encoding a protein of 417 amino acids, has a calculated molecular weight of 47,333.

A putative polyadenylation signal TAATTT is present at the 1,757th nucleotide. The central sequence motif of the polyadenylation signal is TTATTT. The TAATTT sequence is an allowed variant of the human polyadenylation signal sequence [21]. Therefore, we concluded that the sequence TAATTT is a variant of the polyadenylation signal. To our surprise, the amino acid sequence derived from the cDNA did not contain KEDYRN sequence derived from the 47 kDa protein on SDS-PAGE. Instead, the cDNA encodes a protein with high homology to tyrosinase/polyphenoloxidase. The size 47 kDa of the corresponding protein was estimated from electrophoresis as described in the cited reference [14]. This value is the size of the protein before the gene is isolated. On the other hand, the size calculated from the primary structure deduced from the nucleotide sequence is 47,333 as described above. An estimated value from the results of electrophoresis has an error span of at least from 0.1 kDa to 0.2 kDa. The signal peptide sequences estimated from SignalP 4.1 Server (http://www.cbs.dtu.dk/services/SignalP/) is MTSLLTLFVHACILIGVNS. Moreover, secretory signal peptide sequences MTSLLTLFVHACILIGVNS contained in the C-terminal side of the primary structure of PfTy is cut out before it becomes a mature protein. Therefore, the size of the mature protein should be 45,316.

The possible cause of this result that the acquired gene does not code the KEDYRN sequence is as follows. Although the band on the gel appears to suggest that a single protein of 47 kDa was identified, closer inspection indicates that it is possible that two or more proteins were also present with similar molecular masses. Consequently, the antibody prepared using the 47 kDa protein(s) may have been prepared in the presence of multiple antigens. Therefore, two or more antibodies may be present in our study. The expression library was screened by this (these) antibody (antibodies). As a result, we believe that a gene without the sequence KEDYRN was also obtained.

This tyrosinase-like protein, termed PfTy, contains a signal sequence and a copper-binding domain signature (CuA and CuB); however, the epidermal growth factor (EGF)-like domain was absent. Copper-containing proteins can be classified into three groups based on how they coordinate copper. According to the above classification, this tyrosinase-like protein is classified to type-3. Recently, a detailed study on the evolution and functional domains of the genes of type-3 copper proteins has been reported [2]. According to this report, all type-3 copper proteins can be further classified into three subclasses based on the possession of other conserved domains or motifs in addition to two copper-binding sites. The *α* subclass has a signal peptide (SP) at the N-terminal end. The *β*-subclass is localized to the cytoplasm. Therefore, the *β*-subclass only contains the domain of the two copper-binding sites. The *γ*-subclass has a cysteine-rich region with EGF-like domains, which may be involved in protein-protein interactions or dimerization and a transmembrane domain (TM) at the C-terminal end and the signal sequence at the N-terminal end. The novel tyrosinase contains an SP domain in addition to the Cu(A)/Cu(B) domain. There is no cysteine-rich region or a transmembrane domain. Therefore, this tyrosinase-like protein belongs to the *α*-subclass from these indicators, as described in Aguilera et al. [2]. Based on the domain structure, the other three tyrosinases cloned from* P. fucata* mantle also belong to the *α*-subclass.

### 3.3. Comparison of the Active Site Structure

**Figure 2:**
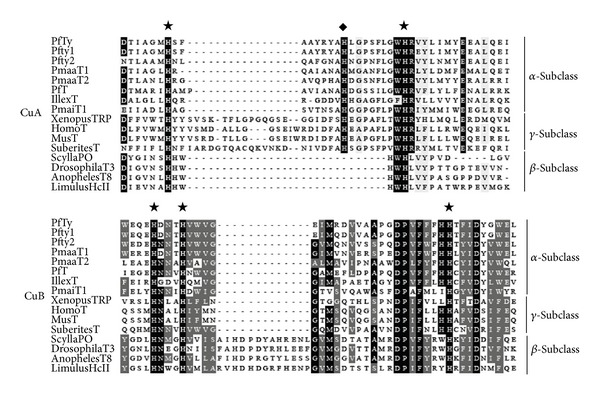
Multiple amino acid sequence alignment of the two putative copper-binding sites. An analysis of sequence alignment of tyrosinase of different species and hemocyanin was performed using the multiple align show (http://www.bioinformatics.org/sms/multi_align.html) server. Five highly conserved histidine residues are indicated by asterisks. The histidine residue present in molluscan tyrosinase is indicated by the diamond. Gaps (_) have been introduced to optimize the alignment. The identical amino acid residues and similar amino acid residues are indicated as black shading and as very light gray shading, respectively. Three subclasses (*α*, *β*, and *γ*) were shown, as described in Aguilera et al. [2]. Abbreviations are as follows and GenBank accession numbers are in parenthesis. PfTy,* Pinctada fucata* tyrosinase-like protein (AB353113); Pfty1,* Pinctada fucata* tyrosinase-like protein 1 (AB254132); Pfty2,* Pinctada fucata* tyrosinase-like protein 2 (AB254133); PmaaT1,* Pinctada margaritifera* tyrosinase 1 (HE610377); PmaaT2,* Pinctada margaritifera* tyrosinase 2 (HE610378); PfT,* Pinctada fucata* tyrosinase (DQ112679); IllexT,* Illex argentinus* tyrosinase precursor 2 (AB107881); PmaiT1,* Pinctada martensii* tyrosinase-like protein tyr-1 (KC870906); Xenopus TRP,* Xenopus laevis* tyrosinase-related protein 1 (NM_001087023); HomoT,* Homo sapiens* tyrosinase (NM_000372); MusT,* Mus musculus* tyrosinase (NM_011661); SuberitesT,* Suberites domuncula* tyrosinase-like protein (AJ574915); ScyllaPO,* Scylla serrata *prophenoloxidase (ABD90511); DrosophilaPO,* Drosophila melanogaster* prophenol oxidase A1 (NP_476812); AnophelesPPO,* Anopheles gambiae* polyphenoloxidase (XM_315074); and LimulusHcII,* Limulus polyphemus* hemocyanin subunit II (AM260213).

The two conserved copper-binding domain signatures (CuA and CuB) of PfTy were compared with those of tyrosinases and POs from other bivalves and various kinds of organisms based on the alignment of the multiple align show program (http://www.bioinformatics.org/sms/multi_align.html). As shown in Figure 2, the CuA copper-binding domain of PfTy was very similar to those of tyrosinases of pearl oysters of the genus* Pinctada* and* Illex argentinus* that belong to the *α*-subclass tyrosinase, but not to the *β*-subclass or *γ*-subclass. However, two histidine residues marked with closed asterisks within the CuA copper-binding domain are conserved in all tyrosinases and* L. polyphemus* HcA examined. The third conserved histidine residue in the molluscan *α*-subclass tyrosinases and *γ*-subclass tyrosinases is also marked with a closed diamond (CuA in Figure 2).

On the other hand, the CuB copper-binding domain of PfTy was very similar to tyrosinases of the genus* Pinctada* and* I. argentinus *that belong to the *α*-subclass and was also similar to those of* Xenopus laevis*,* Homo sapiens*,* Mus musculus, *and the Sponge* Suberites domuncula* that belong to the *γ*-subclass. Three histidine residues marked with closed asterisks within the CuB copper-binding domain are conserved in all subclass enzymes examined including* L. polyphemus* HcA (CuB in Figure 2).

### 3.4. Phylogenetic Analyses

**Figure 3:**
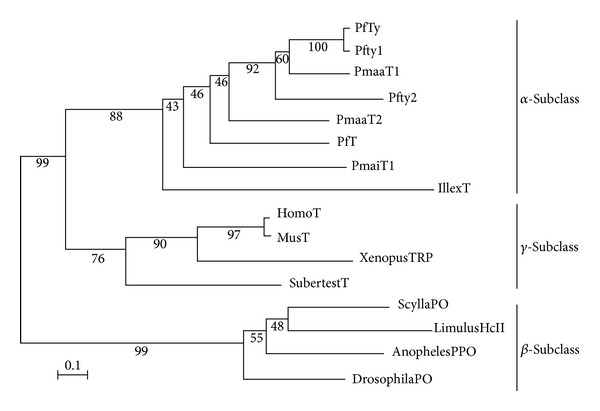
Phylogenetic tree based on the alignment of the CuB copper-binding regions. The phylogenetic tree was constructed by the NJ method as described in Section 2. Numbers at the tree nodes indicate the bootstrap values from 1,000 replicates. The abbreviated name of each protein is the same as presented in Figure 2. The scale bar represents an evolutionary distance of 0.1 amino acid substitutions per protein.

To reveal the evolutionary relationships of these enzymes and hemocyanin, a phylogenetic analysis was performed as described in Section 2. As shown in Figure 3, PfTy was phylogenetically very close to genus* Pinctada *and* I. argentinus* tyrosinases belong to the same clade. The *α*-subclass and *γ*-subclass split from the same node, suggesting that these subclasses have a common ancestor. Moreover, the *β*-subclass belongs to another clade. These results were very similar to previous results [2, 5, 13]. Interestingly, the relation of these three classes was also the same in the phylogenetic tree made from the whole amino acid sequence array of each tyrosinase and hemocyanin despite having other domains in addition to the CuB binding region (data not shown). Moreover, we have obtained almost the same phylogenetic tree result when we made the analysis based on the alignment of the CuA binding domain (data not shown). Therefore, it is assumed that the structure of the CuA and CuB binding domains reflects the overall structure of the protein of each class.

### 3.5. In Vitro Transcription/Translation and Assay of Tyrosinase Activity

**Figure 4:**
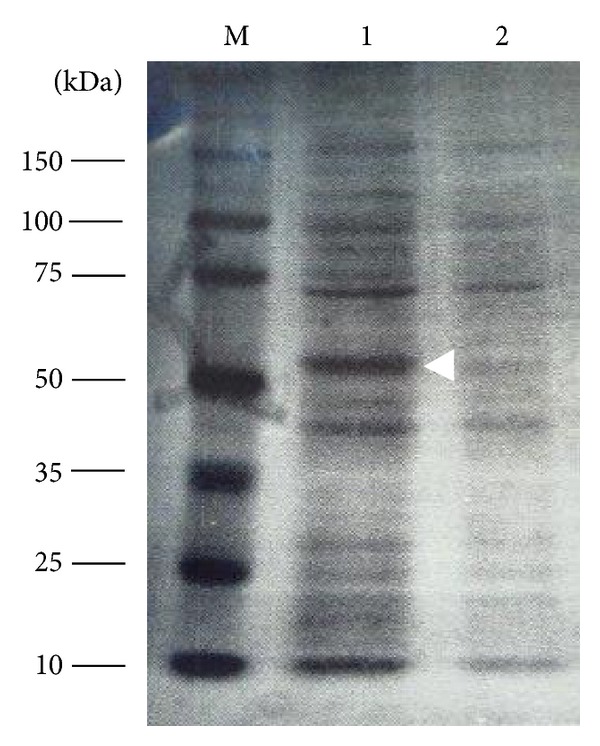
SDS-PAGE analysis of the in vitro transcription-translation product. The pET-29b vector containing the full-length tyrosinase-like gene cDNA was transcribed from a T7 promoter and translated, as described in Section 2. Translation products were subjected to 10% SDS-PAGE as described in Section 2 and visualized by Coomassie brilliant blue R-250. Lane M, perfect protein markers (Novagen): lane 1, about 20 *μ*g of translation product of the transcript from the pET-29b vector containing the full-length tyrosinase-like gene cDNA: lane 2, about 20 *μ*g of translation product of the transcript from the pET-29b vector only. The white triangle indicates the 52 kDa tyrosinase-S-Tag fusion protein.

**Figure 5:**
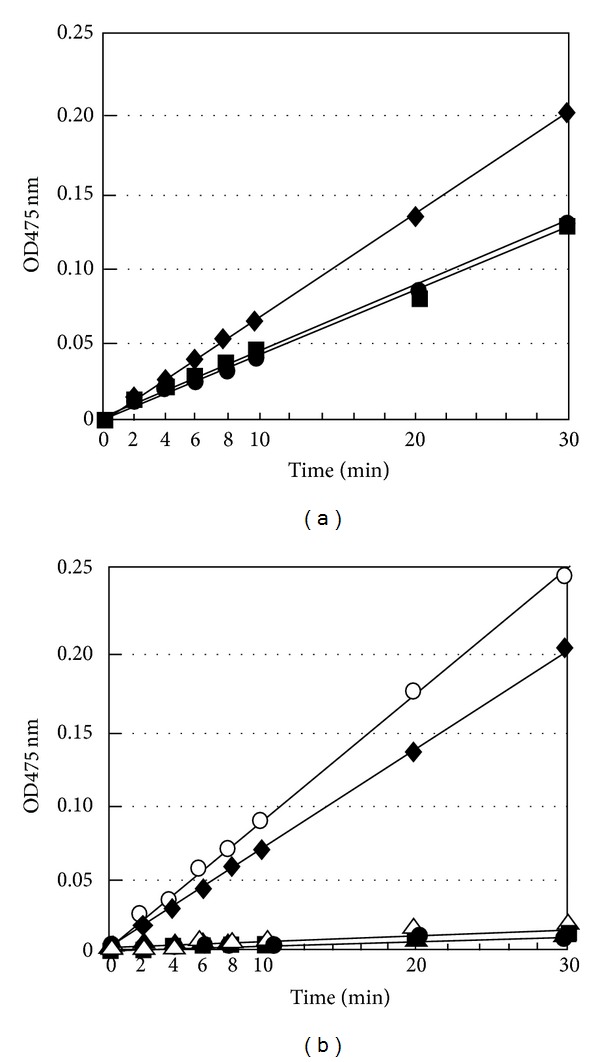
Measurement of the enzyme activity of the in vitro translation product and mushroom tyrosinase. The spectrophotometric tyrosinase assay at 475 nm was performed as described in Section 2. (a) In the absence of EDTA; the translation product of pET29b-Tlg-cDNA (*◆*-*◆*), the translation product of pET29b (■-■), blank (●-●). (b) Mushroom tyrosinase (○-○), the translation product of pET29b-Tlg-cDNA (*◆*-*◆*), mushroom tyrosinase + EDTA (△-△), the translation product of pET29b-Tlg-cDNA + EDTA (▲-▲), the translation product of pET29b + EDTA (■-■), blank + EDTA (●-●). Blank is a 500 *μ*L solution of the substrate (L-DOPA). Abbreviations are as follows: Tlg-cDNA, tyrosinase-like gene cDNA. The used amount of mushroom tyrosinase was 0.1 unit (0.1 *μ*g) dissolved in the 20 *μ*L of H_2_O.

In this study, we have employed cell-free systems derived from* E. coli* to produce the tyrosinase protein [22]. First, the* Eco*RI-*Hind*III tyrosinase cDNA fragment was cloned into the same site of the pET29b vector and subsequent to the T7 phage promoter. The construct was used in the in vitro expression system coupled transcription/translation by using the S30 T7 high-yield protein expression system, as described in Section 2. The tyrosinase was expressed as an S-Tag fusion protein. The expressed fusion tyrosinase protein was analyzed by SDS-PAGE, and then enzymatic activity was probed by a zymogram as described in Section 2. The expressed fusion protein was *≈*52 kDa including the *≈*0.5 kDa portion of the S-Tag sequence in addition to the extra amino acid sequence present in front of the* Eco*RI site and was confirmed clearly on the gel (Figure 4, open triangle). However, we failed to detect enzyme activity of the in vitro translated protein by zymography. The enzyme activity of the mushroom tyrosinase could also not be detected by the zymography method when using 0.1 *μ*g of material; however, activity was detected by the spectrophotometric method, which uses an equivalent amount of material, as described in the legend of Figure 5 (data not shown). Therefore, we performed a spectrophotometric tyrosinase assay. Dopachrome is one of the products of the tyrosinase-catalyzed oxidation and has a peak absorbance at 475 nm. The spectrophotometric tyrosine assay at 475 nm was performed, as described in Section 2. The reaction was started by the addition of the in vitro translation product. As shown in Figure 5, the plot of absorbance at 475 nm versus time shows the relation between the amount of the reaction product dopachrome (*y*-axis) produced and the reaction time (*x*-axis). In Figure 5(a), the absorbance at 475 nm progressed linearly up to 30 min in the presence of the translation product of pET29b-Tlg-cDNA (*◆*-*◆*). The increase of this absorption value was also observed for the translation product of pET29b, which does not include a tyrosinase-like gene (■-■) and also for the blank reaction that contains only substrate L-DOPA (●-●). Nonetheless, the slopes for these two latter reactions were not as steep as observed for the reaction with the expressed translation product. The reason for the increase in the absorption values is the natural oxidation of the substrate L-DOPA. Thus, this nonenzymatic increase in dopachrome was due to oxidation of L-DOPA. Therefore, reactions were performed under the existence of an antioxidant substance, EDTA. The results are presented in Figure 5(b). The absorbance at 475 nm progressed linearly up to 30 min in the presence of the translation product of pET29b-Tlg-cDNA as observed in Figure 5(a) (*◆*-*◆*). The increasing absorbance was also observed in the presence of mushroom tyrosinase (○-○). However, the optical density increase was not observed in the reaction containing the translation product of pET29b-Tlg-cDNA (▲-▲) or mushroom tyrosinase (△-△) when EDTA was present. Thus, Cu ions are essential for the tyrosinase-catalyzed reaction. EDTA is a powerful and commonly used chelating agent. EDTA removes heavy metals from reaction solutions. Therefore, tyrosinase does not function in the presence of EDTA. On the other hand, the optical density increase was not observed in the reaction containing the translation product of pET29b (■-■) in the presence of the antioxidant (i.e., EDTA). The optical density increase was also not observed in the blank reaction containing L-DOPA and EDTA (●-●). Dopachrome was not produced since the antioxidant substance inhibited the nonenzymatic oxidation of L-DOPA. The results described showed that the protein product of the cloned gene expressed in the cell-free system is a tyrosinase.

The reason that tyrosinase activity was not detected by the zymography is thought to be as follows.* * Tyrosinases are activated by various kinds of posttranslational modifications such as serine protease processing [23], glycosylation [24], phosphorylation [25], and disulfide bond formation [26]. Additionally, there may be some other reasons such as presence of SDS that destroys disulfide bond formation and conformation in the zymography. Therefore, it is assumed that the tyrosinase activity observed was insufficient to be detected by zymography that has a considerably lower sensitivity to detect the enzyme activity than the spectrophotometric method as described in the first half in this chapter.

The specific activity of two tyrosinases was next described. The specific activity of mushroom tyrosinase is 1000 unit/mg, because the enzyme material used was 0.1 unit/0.1 *μ*g, as described in the legend of Figure 5. On the other hand, the specific activity of the in vitro translated protein (the S-tag fusion 47 kDa protein) was calculated as follows. Staining intensity of the S-tag fusion 47 kDa protein (arrow) is identical to the marker band of 75 kDa presented in Figure 4 (lane M versus lane 2). In view of the width of the band of the stained protein, there is the possibility that the staining intensity of the S-tag fusion 47 kDa protein has the same grade of staining intensity as the marker proteins of 50 kDa. The quantity of the size marker proteins at 75 and 50 kDa is 0.5 and 1.0 *μ*g, respectively. Therefore, the quantity of the S-tag fusion 47 kDa protein is also presumed to be between 0.5 and 1.0 *μ*g. In Figure 5(b), the absorption value of the mushroom tyrosinase is about 1.25 times the absorption value of the in vitro translated protein (S-tag fusion 47 kDa protein) from the relationship between the absorption value and a reaction time of each of the two enzymes. Therefore, the specific activity of the in vitro translated protein (S-tag fusion 47 kDa protein) is between 80 and 160 unit/mg, because the specific activity of the mushroom tyrosinase is 1000 unit/mg.

### 3.6. Gene Expression Analysis of the Tyrosinase Gene* PfTy*

**Figure 6:**
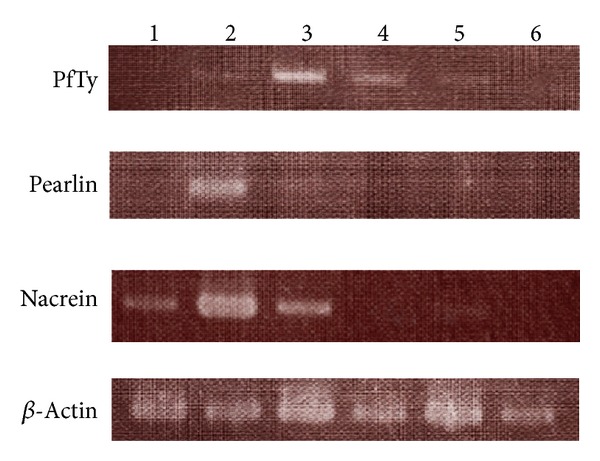
RT-PCR analysis of PfTy and some shell matrix protein genes expressed in* Pinctada fucata*. Lane 1, the mantle edge; lane 2, the mantle pallial; lane 3, liver; lane 4, adductor muscle; lane 5, foot; and lane 6, gill.

Four tyrosinase genes have already been reported, including the* PfTy* gene of this paper in* P. fucata*. These are Pfty1, Pfty2 [13], and* P. fucata* tyrosinase [2]. These are highly homologous to each other. However, the terminal DNA sequence of the 3′ flanking region of the PfTy gene reported here does not exist in the other three genes. To detect the expression of the PfTy gene specifically by RT-PCR analysis, two primers were designed within the arrangement of the 3′ flanking region, as described in Section 2. A previous report showed that* P. fucata* tyrosinase and Pfty2 were transcribed in the outer edge and in the inner part (pallial) of the mantle, respectively [2, 13]. In these sites of the mantle, the former corresponds to the formation of the prismatic calcite shell layer and the latter corresponds to the formation of the nacreous aragonite shell layer, respectively. Moreover, Pfty1 was transcribed in both layers [13]. The control genes used in this analysis were the Cys-rich nacreous layer-specific protein pearlin [15], which is a homolog of N16 [27] and is transcribed only in the pallial of the mantle tissue [28], whereas the carbonic anhydrase nacrein [19] is transcribed in both tissues [28] and in some other tissues [29]. In this report, the expression profile of these control genes in the mantle was successfully reproduced (Figure 6).

RT-PCR analysis showed that PfTy was transcribed in the mantle pallial, but not in the mantle edge. These results were also observed for Pfty2 [13]. As discussed in Section 1, tyrosinase has various functions in addition to melanin synthesis. Therefore, as with Pfty2, PfTy participates in the insoluble shell matrix formation of the nacreous layer. PfTy was also transcribed in several tissues such as foot, liver, adductor muscle, and gill. These results suggest that PfTy also participates in the synthesis of melanin, which is an effective scavenger of free radicals formed in multiple intracellular oxidative processes [30, 31].

## 4. Conclusions

Tyrosinases play an important role in the formation of the shell matrix and melanin synthesis in mollusks shells. A cDNA clone encoding a 47 kDa protein was isolated from the pearl oyster* P. fucata*. The cDNA is 1,957 base pairs long and encodes a protein that is 417 residues in length, and this protein has a clear identity with tyrosinase (polyphenol oxidase; EC 1.14.18.1). This tyrosinase-like protein, termed PfTy, contains an N-terminal signal sequence and two copper-binding domain signatures (CuA and CuB), suggesting that PfTy belongs to the *α*-subclass. The enzyme activity of PfTy was examined by a spectrophotometric method using the translation product derived from an S30 T7 high-yield protein expression system. Tyrosinase activity was identified from this analysis. RT-PCR analysis showed that PfTy mRNA was expressed in the mantle pallial, but not in the mantle edge. Therefore, PfTy participates in insoluble shell matrix formation of the nacreous layer. The expression of PfTy was also observed in the foot, liver, and adductor muscle, suggesting that PfTy takes part in the synthesis of melanins, which are effective scavengers of free radicals formed in multiple intracellular oxidative processes. This is the first report of a novel *α*-class tyrosinase from the pearl oyster* P. fucata*.
